# Evaluation of meibomian gland dysfunction in type 2 diabetes with dry eye disease: a non-randomized controlled trial

**DOI:** 10.1186/s12886-023-02795-7

**Published:** 2023-01-31

**Authors:** Qin Yang, Lihua Liu, Jing Li, Hui Yan, Haiying Cai, Minjie Sheng, Bing Li

**Affiliations:** Department of Ophthalmology, Yangpu Hospital, Tongji University School of Medicine, 450 Tengyue Road, Shanghai, 20090 China

**Keywords:** Dry eye disease, Meibomian gland degeneration, Diabetes, Keratograph

## Abstract

**Background:**

The purpose of this investigation was to evaluate the morphology and physiological function of the meibomian glands between type 2 diabetics with dry eye disease (DED) and control subjects. Doing so will help to better reveal the pathologic mechanisms of meibomian gland dysfunction (MGD) and DED in type 2 diabetes mellitus (T2DM).

**Methods:**

Ninety subjects were divided into the following four groups: DM-DED group: T2DM patients with DED (*n* = 30); DM control group: DM patients without DED (*n* = 18); DED group: DED patients without DM (*n* = 26); and normal control group: normal subjects (*n* = 16). All participants administered the ocular surface disease index (OSDI) questionnaire, tear meniscus height (TMH), noninvasive Keratograph tear film break-up time (NIKBUT), Schirmer I test (SIT), corneal fluorescein staining (CFS), eyelid margin abnormality examinations, meibum quality and meibomian gland (MG) dropout evaluations.

**Results:**

The percentage of MG dropout in the upper and lower lids was significantly higher in the DM-DED group than the DED group (*P* < 0.05 or *P* < 0.01). However, there was no significant difference in other MG parameters between these two groups. Oppositely, Significant difference was observed in all of MG parameters except MG dropout in the lower lids comparing DM group with normal controls (*P* < 0.05 or *P* < 0.01). While the SIT values decreased in the DM-DED group compared to the DED group (*P* < 0.05), no significant differences were found in the values of other tear parameters.

**Conclusions:**

The higher prevalence and increased severity of MGD was found in patients with both T2DM and DED compared to those only with DED.

**Trial registration:**

Chinese Clinical Trial Registry ChiCTR1800019939, date of registration December 9, 2018, prospectively registered.

## Introduction

Type 2 diabetes mellitus (T2DM) is a serious public health problem worldwide, which can cause numerous ocular complications, such as diabetic retinopathy, cataract, punctuate keratopathy, and dry eye disease (DED). The incidence of DED in diabetes was reported at 54% [1].

The meibomian gland, a special sebaceous gland, may also be targeted in patients with T2DM which is a systemic metabolic disease linked with lipid overload and hyperlipidemia. It was suggested that diabetes was a risk factor of asymptomatic meibomian gland dysfunction (MGD) in a large cohort epidemiologic study [2]. MGD, which is the most prevalent cause of the evaporative dry eye, can change the quality and quantity of the lipids in meibomian gland secretions [3, 4]. The abnormal expression of meibum lipids causes the loss of homeostasis in the tear film and leads to the development of DED.

Previous studies have demonstrated that MGD in patients with T2DM was more severe compared to MGD in nondiabetics [5–8]. However, it is necessary to prove the consistency of the results in these studies, as there are numerous grading subdivisions for each sign in the evaluation of MGD. What’s more, it would be more objective to compare diabetics with DED to nondiabetic patients with DED. Therefore, in this study, we investigated the morphology and physiological function of meibomian gland in T2DM patients with DED to further reveal the relationship between DED and MGD in T2DM.

## Methods

### Subjects

This cross-sectional study is part of the clinical trial: the Research for the Morphology, Function and Lipids of Meibomian Glands in Diabetic Patients with Dry Eye, conducted in Department of Ophthalmology at Yangpu Hospital of Shanghai from December 2018 to December 2019. The protocol was registered in the Chinese Clinical Trial Registry (09/12/2018, ChiCTR-180001939) and approved by the medical ethics committee of the Yangpu Hospital, Tongji University School of Medicine (Shanghai, China) in accordance with the WMA Declaration of Helsinki. Written informed consent was obtained from each participant at the examination site. The eligible criteria and grouping process were reported in the previous paper [9].

**Fig. 1:**
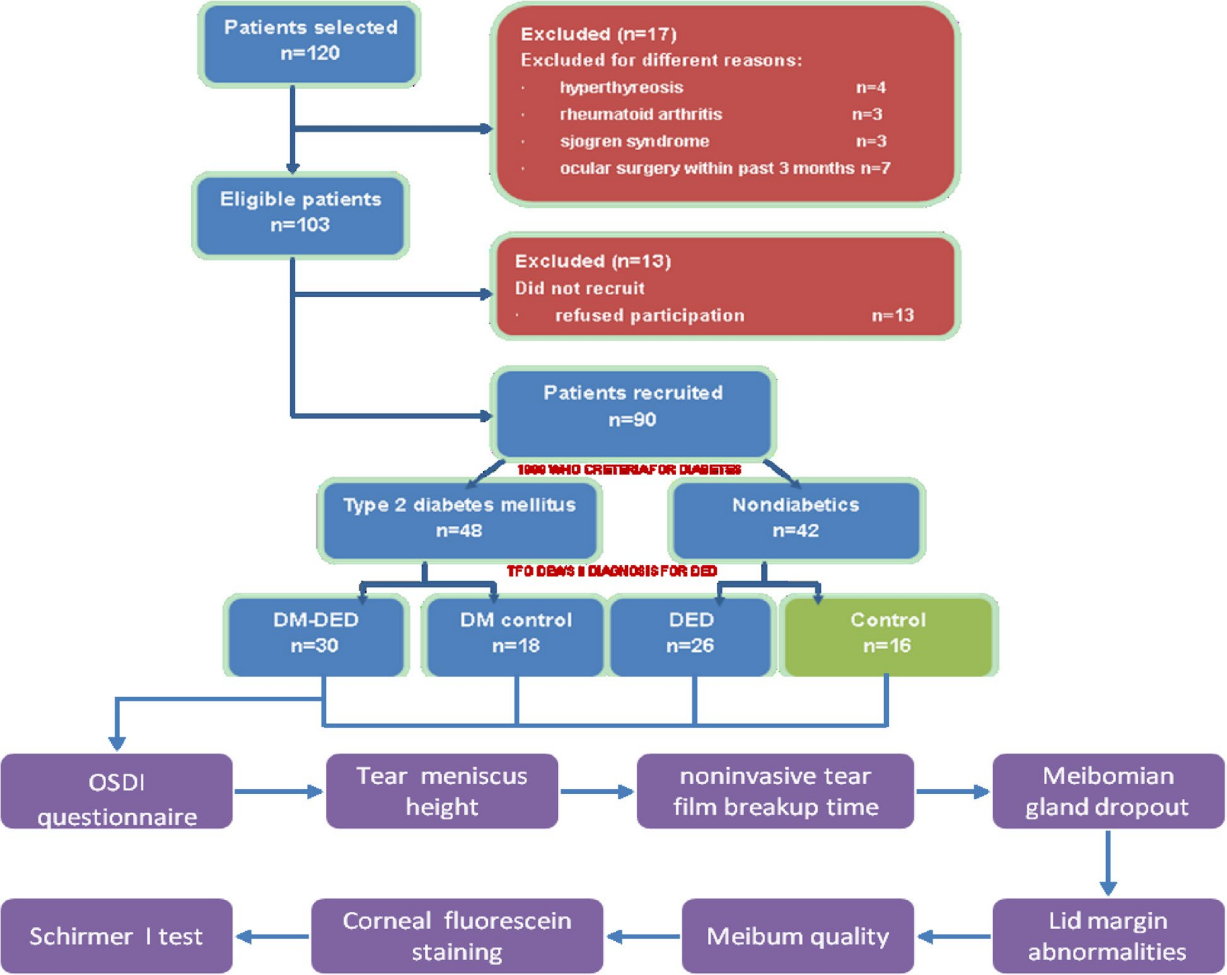
Flow chart illustration of the study procedure (DM, diabetes mellitus; DED, dry eye disease)

The inclusion criteria were as follows: 1) patient was at least 40 years old; 2) all assessments that qualify in the right eye to minimize the influence factors to the results; 3) any patient with T2DM fulfilled the criteria established by the WHO in 1999, with diabetes duration of at least five years for early diabetes has no or mild systemic effects; 4) any patient with DED met the criteria set out by the DEWS II in 2017; and 5) patient had a willingness to comply with the study protocol. The patients with following conditions were excluded from the study: 1) active eye inflammation and infection within the past three months; 2) use of any eye drops including artificial tears within the past three months; 3) wearing contact lenses within the past three months; 4) ocular laser surgery or other surgery and ocular trauma within the past three months; 5) other systemic disease conditions that cause dry eye, particularly autoimmune disease. A minimum sample of 24 patients per group was calculated by PASS software (a two-sided *α* = 0.05, β = 0.2, δ (expected difference) = 0.17, and σ (standard deviation) = 0.2). Assuming an expected loss rate of 20%, each group included 30 patients. Ninety Chinese patients between 41 and 82 years of age were enrolled after thirty patients dropped out the study for various reasons, and were divided into following four groups (Fig. 1): DM-DED group: T2DM patients with DED (*n* = 30); DM control group: DM patients without DED (*n* = 18); DED group: nondiabetic patients with DED (*n* = 26); control group: normal subjects (*n* = 16). Those performing examinations or accessing outcomes were blinded for assignment of patients.

### Clinical examinations

All the tests were performed from the least to the most invasive. So lid margin assessment and corneal fluorescein staining were completed before meibography.

#### Ocular symptoms assessment

All patients completed the ocular surface disease index (OSDI) questionnaire. It contains three subsections, including vision-related function, ocular symptoms, and environmental triggers. OSDI scores range from 0 to 100, with 0–12 representing normal, 13–22 representing mild DED, 23–32 representing moderate DED, and higher than 33 representing severe DED [10].

#### Noninvasive ocular surface examinations

The Oculus Keratograph 5 M (K5M, Wetzlar, Germany) was used for all patients in the following order: tear meniscus height (TMH), noninvasive Keratograph tear film breakup time (NIKBUT), and meibomian gland (MG) dropout.

#### TMH

TMH was measured three times in the right eye using infrared images obtained from the Keratograph, and the average of TMH was used in analysis. The lower tear film meniscus images were captured after blinking twice, and the values were generated vertical to the central lower eyelid margin [11].

#### NIKBUT

NIKBUT was measured twice in the right eye using the noninvasive K5M tear breakup time tool. The subjects were instructed to blink twice before screening and to keep their eyes open to the best of their ability when recording. The first NIKBUT (NIKBUT-1st) and NIKBUT average (NIKBUT-avg) were then automatically generated. The NIKBUT-1st indicates the time at which the tear film begins to break up. The NIKBUT-avg represents the average time at which the overall tear film breaks up [5].

#### MG dropout

**Fig. 2:**
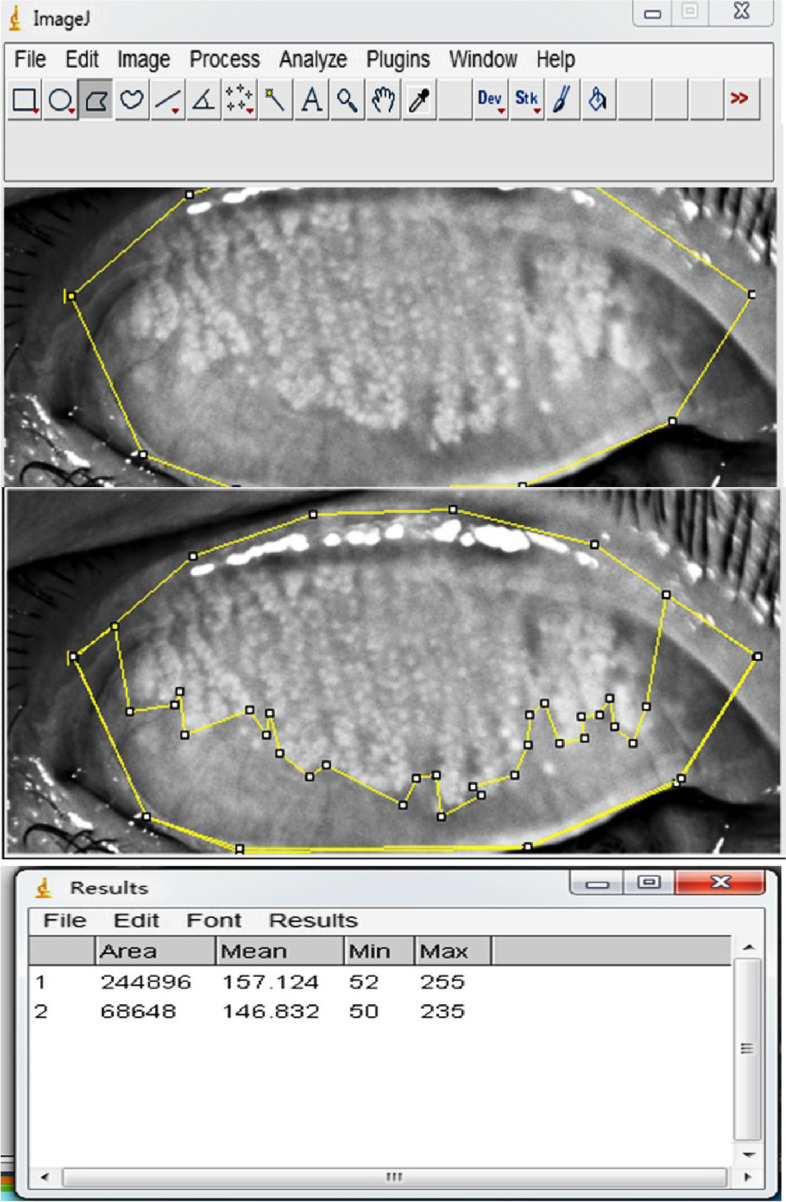
Analyses of the area of meibomian gland loss using Image J software

Following the standard operating procedure for the K5M, the upper and lower eyelid of right eye of the subjects were upturned to expose the palpebral conjunctiva to obtain images of the MGs. Images were analyzed with semi-automatic Image J software (https://imagej.nih.gov/ij/, National Institutes of Health, Bethesda, MD, USA) by a specialized observer. The percentage of MG dropout was calculated using a technique initially described by Pult [12] (Fig. 2).

#### Lid margin abnormalities

Lid margin findings were assessed according to the following four signs: vascular engorgement (absence 0, presence 1), lid margin irregularity (absence 0, presence 1), obstructed meibomian gland orifices (absence 0, presence 1), and anterior or posterior displacement of the mucocutaneous junction (absence 0, presence 1). The lid margin score was recorded from 0 to 4 [13]. Both the upper and lower eyelids were evaluated with the use of a slit-lamp microscope (HANG-STREIT, Switzerland).

#### Meibum quality

MGs secrete meibum, which contains components of the lipid layer of the tear film. The quality of meibum was assessed for the upper and lower eyelids according to the grading schemes: 0, clear (normal); 1, cloudy; 2, cloudy with particles; and 3, inspissated (like toothpaste). A score was recorded only the highest grade encountered from any of central eight glands, and the score range was 0 to 3 [14].

#### Corneal fluorescein staining (CFS)

The fluorescein punctate staining of the cornea was evaluated using the grading scheme of the National Eye Institute/Industry Workshop guidelines [15]. Briefly, the cornea was divided into five sectors (central, superior, inferior, nasal, and temporal), and each sector was scored from 0 to 3.The total score was determined out of 15.

#### The Schirmer I test (SIT)

The Schirmer test was performed without topical anesthesia. The folded end of the standard paper strip was placed in the temporal one-third of the lower lid margin. The length of each wetting was measured and recorded after a period of five minutes, whence the subject was instructed to close the eye [16].

### Statistical analysis

All statistical analyses were performed using SPSS Statistics version 22.0 (IBM Corp., Armonk, NY, USA). All data are presented as the mean ± standard. The chi-square test was used to compare sex and age among the four groups. The means of the parameters (OSDI scores, TMH, NIKBUT, FL, lid margin abnormality, MG dropout, meibum quality, and SIT) were compared among the groups with one-way ANOVA or Welch ANOVA according to the homogeneity of variance. The Spearman correlation test was used to calculate correlations. All *P* values less than 0.05 were considered statistically significant.

## Results

### Summary of general information

Ninety subjects were included in this study. In 30 diabetic patients with DED, there were 15 men, and the mean age was 64.46 ± 9.29 years; the participants’ mean duration of diabetes (from diagnosis) was 12.15 ± 7.45 years. The T2DM patients without DED included 11 men and 7 women (aged 61.11 ± 6.58 years), whose mean duration of diabetes (from diagnosis) was 12.83 ± 6.71 years. There were 26 nondiabetic patients with DED (10 men and 16 women, aged 63.58 ± 8.56 years); 16 normal subjects (7 men and 9 women) were recruited as controls with a mean age of 60.06 ± 7.18 years. The demographic and clinical characteristics of the participants are summarized in Table 1. Age and gender did not differ significantly among the subject groups.

**Table 1:** Clinical characteristics of study subjects

Characteristics	DM-DED	DM control	DED	Control
Age(years)	64.46 ± 9.29	61.11 ± 6.58	63.58 ± 8.56	60.06 ± 7.18
Sex ratio(male/female)	15/15	11/7	10/16	7/9
DM duration(years)	12.15 ± 7.45	12.83 ± 6.71	/	/
FBG(mmol/L)	8.66 ± 2.63	8.31 ± 2.32	/	/

*DED* dry eye disease, *DM* diabetes mellitus, *FBG* fasting blood glucose

### Comparison of Meibomian gland parameters among groups

Table 2 shows the parameters of tears and meibomian glands in the DM-DED, DM control, DED, and normal groups. Table 3 displays the results of the comparison of these parameters among the four groups.

**Table 2:** Characteristics of tear and meibomian gland in study groups

Characteristics	DM-DED	DM control	DED	Control
OSDI	16.63 ± 10.62	6.17 ± 6.07	19.61 ± 11.05	11.21 ± 8.21
TMH(mm)	0.22 ± 0.06	0.28 ± 0.08	0.20 ± 0.06	0.27 ± 0.07
SIT(mm)	6.65 ± 3.46	9.78 ± 2.90	9.70 ± 3.67	14.0 ± 5.98
NIKBUT-1st(s)	3.47 ± 0.99	12.29 ± 6.37	3.49 ± 1.14	14.84 ± 5.57
NIKBUT-avg(s)	4.15 ± 0.98	14.34 ± 5.97	4.37 ± 2.07	16.80 ± 4.74
CFS	3.46 ± 2.44	1.02 ± 2.30	3.18 ± 2.01	0.31 ± 0.60
Lid margin abnormality score				
Upper	2.75 ± 0.93	2.06 ± 0.99	2.19 ± 0.75	1.13 ± 0.50
Lower	2.32 ± 1.12	1.72 ± 0.67	1.65 ± 0.56	0.56 ± 0.69
Meibum score	2.21 ± 0.63	1.89 ± 0.67	1.85 ± 0.73	0.69 ± 0.60
MG dropout(%)				
Upper	33.47 ± 8.24	21.17 ± 7.24	23.92 ± 10.48	16.46 ± 6.59
Lower	36.97 ± 10.67	28.75 ± 12.12	29.05 ± 12.13	16.80 ± 4.74

*OSDI* ocular surface disease index, *TMH* tear meniscus height, *NIKBUT* noninvasive tear film breakup time, *MG* meibomian gland, *DED* dry eye disease, *DM* diabetes mellitus

**Table 3:** Comparison (*P* values) of characteristics of tear and meibomian gland among study groups

Characteristics	DM-DED vs DED	DM-DED vs Control	DM control vs Control
OSDI	0.252	0.046*	0.147
TMH(mm)	0.188	0.005*	0.666
SIT(mm)	0.028*	0.011*	0.022*
NIKBUT-1st(s)	0.99	0.001*	0.057
NIKBUT-avg(s)	0.812	0.002*	0.052
CFS	0.663	0.039*	0.336
Lid margin abnormality score			
Upper	0.190	0.001*	0.009*
Lower	0.251	0.002*	0.005*
Meibum score	0.096	0.003*	0.006*
MG dropout(%)			
Upper	0.004*	0.001*	0.214
Lower	0.021*	0.004*	0.016*

*OSDI* ocular surface disease index, *TMH* tear meniscus height, *NIKBUT* noninvasive tear film breakup time, *MG* meibomian gland, *DED* dry eye disease, *DM* diabetes mellitus

^*^P < 0.05

**Fig. 3:**
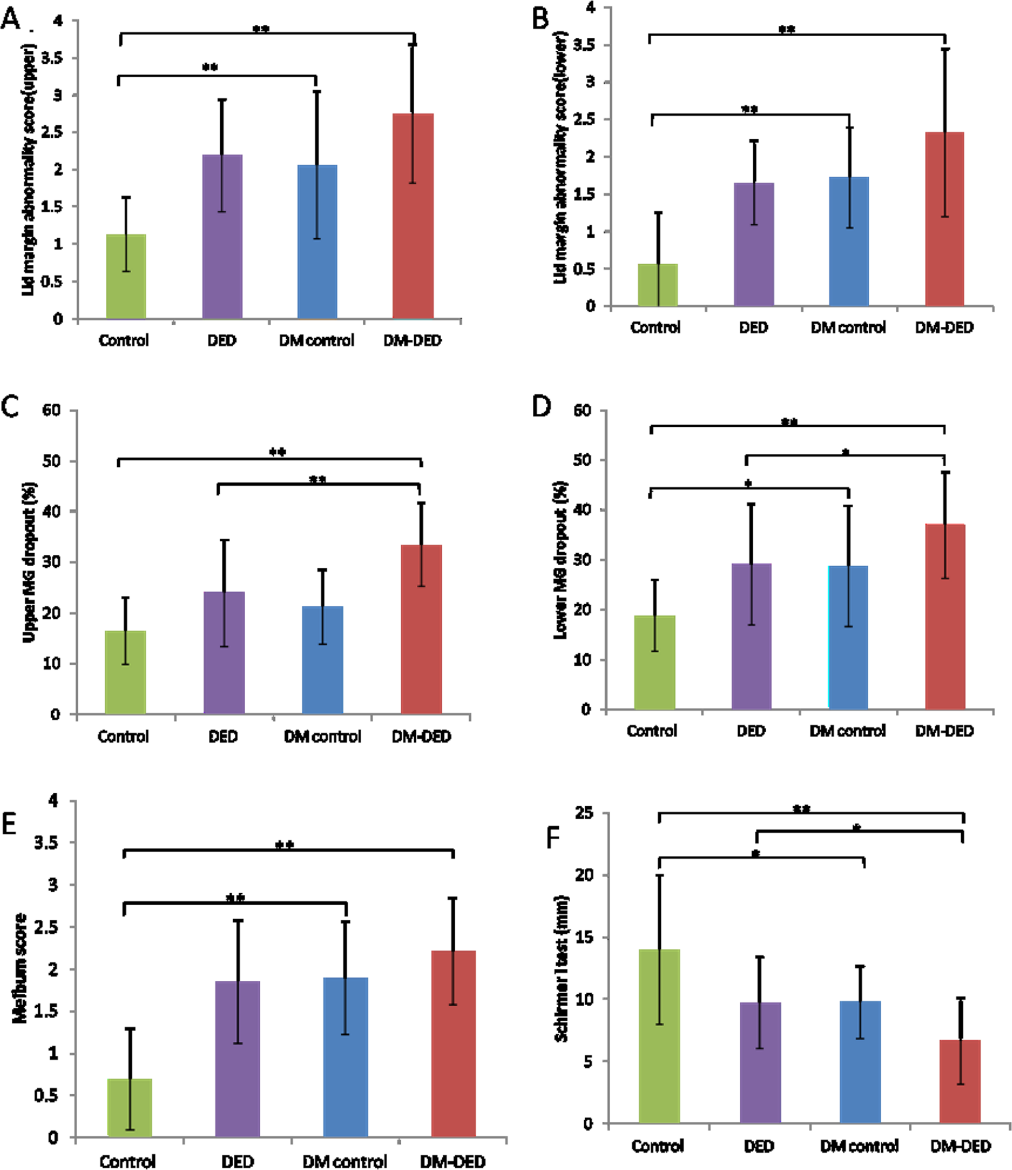
Comparisons of parameters of tear and meibomian gland among study groups. **A** Lid margin abnormality sore (upper); **B** Lid margin abnormality sore (lower); **C** Upper MG dropout; **D** Lower MG dropout; **E** Meibum score; **F** Schirmer I test (mm); MG, meibomian gland; DED, dry eye disease; DM, diabetes mellitus, *P < 0.05; **P < 0.01

The mean values of both upper- and lower–meibomian gland loss (33.47% ± 8.24%, 36.97% ± 10.67%) were significantly higher in the DM-DED group than in the DED patients (23.92% ± 10.48%, 29.05 ± 12.13%) and the normal controls (*P* < 0.01; Table 3, Fig. 3). Similarly, higher loss of both upper and lower meibomian glands were shown in the DM controls (21.17% ± 7.24%, 28.75% ± 12.12%) compared to the normal controls, but significant difference was found only in the lower meibomian gland (*P* = 0.021; Table 3, Fig. 3). Additionally, the meibum quality score was significantly higher in both the DM-DED and DM controls compared to the normal controls (*P* < 0.01; Table 3, Fig. 3). The lid margin abnormality scores of both the upper and lower meibomian glands were also significantly higher when comparing the DM-DED and DM patients to the normal controls (*P* < 0.01; Table 3, Fig. 3). There were no significant differences in lid margin abnormality or meibum quality scores of both the upper and lower meibomian glands between the DM-DED and DED groups.

### Comparison of tear parameters among groups

Significantly, differences were observed in the mean values of the OSDI, NIKBUT-1st, NIKBUT-avg, TMH, SIT, and CFS between the DM-DED and normal control groups (Table 3). Additionally, the value of the SIT was significantly lower in the DM-DED group compared to the DED group without DM (*P* < 0.01; Table 3, Fig. 3); however, there were no differences in the values of other tear parameters between these two groups. Similarly, significantly lower SIT values were observed in the DM control group compared to the normal control group (*P* < 0.05; Table 3, Fig. 3); no difference was observed in the values of the other tear parameters between these two groups.

### Correlations between clinical parameters in diabetic patients

**Fig. 4:**
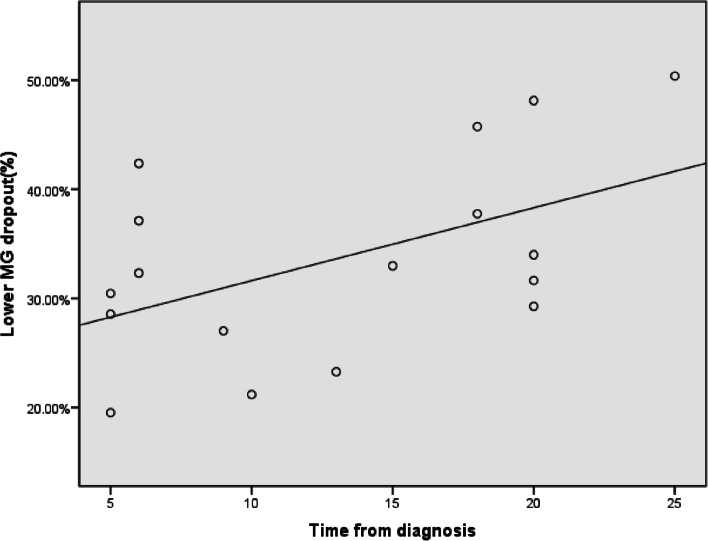
Scatterplot graph of lower meibomian gland dropout and time from diagnosis in the diabetics without dry eye disease

**Fig. 5:**
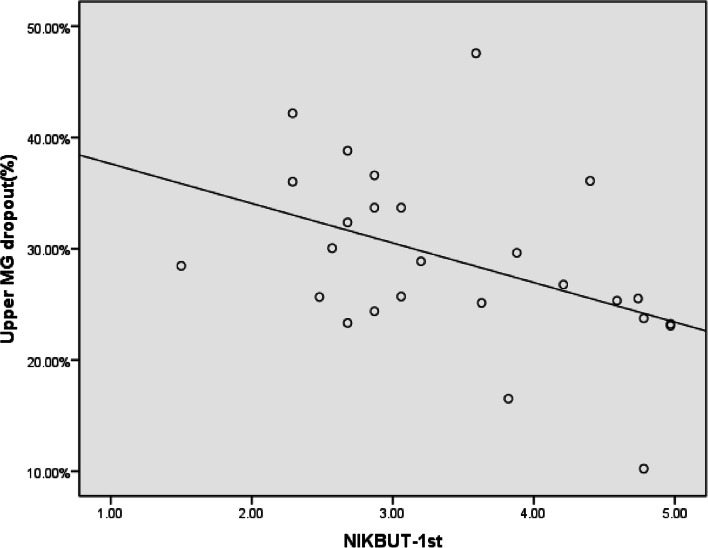
Scatterplot graph of upper meibomian gland dropout and first noninvasive keratograph tear film break time in the diabetics with dry eye disease

Spearman correlation analysis showed that the duration of diabetes was significantly correlated with the lower meibomian gland loss in the DM control group (*R* = 0.509, *p* < 0 05; Fig. 4) but had no correlation with other meibomian gland and tear parameters. We also observed that NIKBUT-1st had a significant negative correlation with the upper meibomian gland loss in the DM-DED group (*r* =  − 0.445, *P* < 0.05; Fig. 5). There was no significant correlation between the other meibomian gland and tear parameters.

## Discussion

Numerous researchers have proven the effects of diabetes on tear film and secretion. However, few studies have focused on the impact of diabetes on MG morphology and function [5–8]. Lin et al. reported that compared to the healthy control group in their study, diabetics showed significantly worse meibomian gland changes, including higher MG dropout, lower number of expressible glands, and higher lid margin abnormality scores [5], which is in agreement with the results of the present study. Moreover, the present authors observed that loss of both upper and lower meibomian glands was notably higher in the DM-DED patients than in the nondiabetic DED patients. Furthermore, significantly lower SIT values were observed in diabetics, but no significant changes in other tear parameters were found. These results suggest that MGD in those with both DM-DED and T2DM is even worse, which can exacerbate DED.

Diagnosis and quantification of MGD involves the evaluation of symptoms (assessed by questionnaires), altered meibomian gland secretions (evaluated by pressing the tarsal plate), changes in lid morphology (assessed by slit-lamp microscopy), and meibomian gland dropout (found by meibography); all of these are characteristics of MGD [17]. Nevertheless there is no standardized procedure for MGD assessment at present, especially in terms of MG secretion and dropout; thus, the consistency of outcomes in clinical trials remains poor. Altered meibomian gland secretion means both the quality and the expressibility of meibum are changed. However, the measurement of meibomian gland expression requires the development of methods to standardize the application of force to the tarsal plate [18–20]. Moreover expression is not in itself a measurement of secretory activity, because it may be difficult to distinguish the glands that are not expressible for physiological reasons or for pathologic reasons [17]. Meibum quality seems to be a more important factor in tear film instability associated with MGD [21], and the assessment of grading schemes is much simpler. Therefore, in the present study, the meibum quality was assessed, rather than the expressibility of the meibum, which was evaluated in Lin’s study [5]. The Keratograph 5 M, a non-invasive commercial technique, can be used to view and photograph the MG dropout within an eyelid using infrared photography. There are many different grading scales available to assess the amount of gland dropout, with a 4- or 5-grade scale being the most widely used in clinical research [17]. Image J has also been used in the semi-automated measurement of the “area of loss of MG” to determine the percentage of MG dropout [22]. Pult reported that better inter- and intra-observer agreement has been shown compared computerized grading with Image J to the subjective 4- or 5-grade scales [23]. Lin and Yu used a 4-grade scale to determinate the meibography scores of diabetics [5, 24]. In the current study, more objectively computerized grading was used to improve the sensitivity and repeatability of the data.

The current investigation focuses on meibomian gland morphology and dysfunction in diabetics with DED and provides more convincing evidence for the correlation between MGD and DM. We observed that the meibomian gland loss was significantly higher in both the upper and lower eyelids of diabetics with DED than in nondiabetics with DED. Although the score of either lid margin abnormality or meibum quality was no correspondingly significant higher, but these two variables are just indirectly reflection of MGD. After all, in early or nonobvious MGD, there are no obvious morphological lid margin changes observed by physical examination [25, 26]. Recently, a study observed the nonobvious MGD subtype with severe MG loss had normal lipid secretion [27]. The authors speculated that few but active MGs could maintain meibum expression. This explanation might apply to the DM-DED group in the current study. Regardless, meibomian gland loss on its own, which can be directly detected, suggests meibomian gland function is surely impaired. Therefore, these results indicated more severely impaired function of the meibomian gland in diabetics with DED than in nondiabetics with DED.

Inconsistent with Lin’s study [5], the loss of MG in the upper and lower eyelids differed compared the DM group to the normal controls in present study. Significant differences were observed only in the lower eyelid, which consistently worsened with increased course of disease; however, this was not the case in the upper eyelid. This discrepancy may be caused by the different methods of assessment of meibomian gland dropout. Alternatively, it indicates that MGD in diabetics may progress earlier in the lower eyelids than the upper eyelids, as there was greater loss of dropout in the lower eyelids compared to the upper lids in the DM and DM-DED groups. Previous studies have reported similar results in nondiabetic patients with MGD [28, 29]. It is assumed the reason may attribute to more strongly squeeze upper eyelids than do lower eyelids during blinking, which induce to secretion of meibum more easily and continuously.

Thus far, the mechanism of MGD induced by DM is unclear, and further research should be performed to verify some speculations. It has been postulated that type 2 diabetes mellitus might play a key role in MGD through stopping the proliferation of human meibomian gland epithelial cells (IHMGECs) and lipid metabolism. Ding’s study found that insulin stimulates IHMGECs, and that glucose exposure is deleterious to them [30]. Moreover, the abnormal expression of meibum lipids was observed in T2DM patients in other research [9]. The authors suggested that these changes in meibum lipids could increase the meibum viscosity, induce periglandular inflammation, promote hyperkeratinization of the terminal ductules, and cause duct obstruction. In addition, inflammation may be identified as a factor resulting in MGD in diabetes. Recently, the inflammatory microenvironment of the MG under diabetic conditions was indicated. It was also found that inflammatory cell infiltration apparently increases and inflammatory cytokine expression was significantly elevated in the MG of DM rats [31].

Our study found no significant differences in most tear parameters between the diabetic and nondiabetic groups, and only the values of Schirmer I test were significantly decreased, whether comparing the DM-DED group with the nondiabetic DED group or the DM control group with the normal control group. Most published research has reported patients with DM have decreased Schirmer test values [32–34]. The factors thought to result in reduced tear production in diabetes are associated with a reduction in lacrimal gland secretions, perhaps due to microvascular damage from hyperglycemia, reduced binding sites for androgens and estrogens [35], autonomic neuropathy [36], and the impairment of corneal sensitivity [37]. In addition, reduced tear production was further confirmed that there was a close relationship between MGD and diabetes, as primary MGD usually has normal Schirmer test results. In contrast, there is significant controversy regarding BUT. Some studies have shown a lower fluorescein BUT and noninvasive BUT [34, 37], while others have suggested no difference [32, 33, 38]. This inconsistency might be attributed to the different inclusion criteria of DM samples. Poor metabolic control, proliferative diabetic retinopathy, and peripheral neuropathy are high risk factors for tear film impairment in diabetes. In this study, some of the patients were ophthalmic outpatients with good metabolic control, and others were inpatients in the department of endocrinology with poor control.

This study also found that the OSDI scores of DM-DED patients were higher than those of the normal controls, but lower than nondiabetic DED patients. Similarly, decreased OSDI scores were shown in the DM control group compared with the normal control group, which was consistent with the study of Lin et al. [5]. The decreased OSDI score may be attributed to the reduced corneal sensitivity, which has been proved association with the reductions of corneal nerve fiber length, density and branch density of diabetes patients observed by in vivo confocal microscopy [39, 40]. It was also shown asymptomatic DED patients with diabetic peripheral neuropathy exhibited clinical signs [41]. These outcomes indicated the symptoms were inconsistent with disease severity of DED in diabetes, and the evaluation of ocular surface sensitivity could help to diagnose DED in this population. Furthermore, preventative treatment should be advised especially during a preoperative examination for cataract or refractive surgery, for example, because these patients are at risk of developing symptomatic DED following the surgery.

The present study had several limitations. First, the small sample size could have affected the results, and the significant correlation between meibomian gland loss of both the upper and lower eyelids and diabetic course should be observed in further studies with a larger sample size. Second, the data of diabetes-related clinical parameters were not perfect. For example, serum glycated hemoglobin (HbA1c), the severity of diabetic retinopathy, and the presence or absence of peripheral neuropathy could all be confounding factors of the results, which should be confirmed in further investigations.

In conclusion, in our study, type 2 diabetics with DED worse MGD than nondiabetic DED patients, with significantly higher MG dropout, as well as non-obvious higher levels of lid margin abnormalities and meibum quality. These findings provide concrete evidence for MGD as related to DM-DED. Nevertheless, it is confusing whether MGD is secondary to DM-DED or the latter is caused by the former. Further study must be performed to reveal the causative link between MGD and DM-DED.

## Data Availability

The datasets used and/or analysed during the current study are available from the corresponding author on reasonable request.

## Abbreviations

T2DM: Type 2 diabetes mellitus
DED: Dry eye disease
MGD: Meibomian gland degeneration
OSDI: Ocular surface disease index
TMH: Tear meniscus height
NIKBUT: Noninvasive Keratograph tear film breakup time
CFS: Corneal fluorescein
